# Preparation, Characterization, and Preliminary In Vitro Testing of Nanoceria-Loaded Liposomes

**DOI:** 10.3390/nano7090276

**Published:** 2017-09-16

**Authors:** Agostina Grillone, Tianshu Li, Matteo Battaglini, Alice Scarpellini, Mirko Prato, Shinji Takeoka, Gianni Ciofani

**Affiliations:** 1 Smart Bio-Interfaces, Istituto Italiano di Tecnologia, Viale Rinaldo Piaggio 34, 56025 Pontedera, Italy; matteo.battaglini@iit.it; 2 Research Organization for Nano & Life innovation, Waseda University, 2-2 Wakamatsu-cho, Shinjuku-ku, 162-8480 Tokyo, Japan; tianshuli@aoni.waseda.jp; 3 The Biorobotics Institute, Scuola Superiore Sant’Anna, Viale Rinaldo Piaggio 34, 56025 Pontedera, Italy; 4 Electron Microscopy Facility, Istituto Italiano di Tecnologia, Via Morego 30, 16163 Genova, Italy; alice.scarpellini@iit.it; 5 Materials Characterization Facility, Istituto Italiano di Tecnologia, Via Morego 30, 16163 Genova, Italy; mirko.prato@iit.it; 6 Department of Life Science and Medical Bioscience, Graduate School of Advanced Science and Engineering (TWIns), Waseda University, 2-2 Wakamatsu-cho, Shinjuku-ku, 162-8480 Tokyo, Japan; takeoka@waseda.jp; 7 Department of Mechanical and Aerospace Engineering, Politecnico di Torino, Corso Duca degli Abruzzi 24, 10129 Torino, Italy

**Keywords:** cerium oxide nanoparticles, liposomes, drug delivery

## Abstract

Cerium oxide nanoparticles (nanoceria), well known for their pro- and antioxidant features, have been recently proposed for the treatment of several pathologies, including cancer and neurodegenerative diseases. However, interaction between nanoceria and biological molecules such as proteins and lipids, short blood circulation time, and the need of a targeted delivery to desired sites are some aspects that require strong attention for further progresses in the clinical application of these nanoparticles. The aim of this work is the encapsulation of nanoceria into a liposomal formulation in order to improve their therapeutic potentialities. After the preparation through a reverse-phase evaporation method, size, *Z*-potential, morphology, and loading efficiency of nanoceria-loaded liposomes were investigated. Finally, preliminary in vitro studies were performed to test cell uptake efficiency and preserved antioxidant activity. Nanoceria-loaded liposomes showed a good colloidal stability, an excellent biocompatibility, and strong antioxidant properties due to the unaltered activity of the entrapped nanoceria. With these results, the possibility of exploiting liposomes as carriers for cerium oxide nanoparticles is demonstrated here for the first time, thus opening exciting new opportunities for in vivo applications.

## 1. Introduction

In cerium oxide, Ce owns the peculiar ability to easily switch between two oxidation states, Ce^3+^ and Ce^4+^, through the loss of electrons and/or oxygen, thus giving origin to crystalline defects or vacancies on the surface of the material [1]. At a nanoscale level, due to the higher surface/volume ratio, this phenomenon is more evident, thus making cerium oxide nanoparticles (nanoceria) more reactive with respect to the bulk material. The discovery of the excellent catalytic activities of nanoceria and their ability to act as free radicals scavengers have opened new perspectives in the biomedical fields and, more specifically, in all those scenarios where oxidative stress plays a crucial role in the pathogenesis of diseases [2]. Previous studies have shown that nanoceria are able to neutralize reactive oxygen species (ROS), including superoxide anions, peroxide radicals, and hydroxyl radicals, by mimicking the activity of enzymes involved in antioxidant defenses such as superoxide dismutase and catalase [3]. The biological activities of cerium oxide nanoparticles were assessed in vitro on several cell culture models such as cardiac [4], neuronal [5], and stem cells [6]; in vivo, nanoceria showed strong antioxidant properties by decreasing both nitric oxide and peroxynitrite formation in a murine model of ischemic cardiomyopathy [7], and they slowed down the progression of retinal degeneration in mouse and rat models [8,9], promoted the regression of retinal vascular lesions in mice [10], and inhibited weight gain in rats [11].

Another peculiar feature of cerium oxide nanoparticles is their ability to act as a pro-oxidant agent at acidic pH values [12]. Several research groups reported that, where tumor microenvironment is acidic, nanoceria triggers ROS accumulation [12], possess cytotoxic and anti-invasive properties [13,14], and sensitize cells to radiotherapy [15]. Anticancer activity of nanoceria was studied in vitro for the treatment of breast, pancreas, ovarian, lung, and colon cancer cells [15,16,17].

Despite the significant promises of nanoceria, several challenges still need to be addressed before a translational exploitation into clinical practice. First of all, nanoceria dispersions are often unstable in aqueous solutions and in high ionic media, quickly precipitating and forming aggregates that hinder their in vivo administration [18] or at least that heavily affect their transport and biodistribution [19]. The interaction of nanoparticles with proteins moreover triggers recognition by phagocytic cells, thus inducing retention in the reticuloendothelial system (mainly in the spleen and in the liver), with a consequent reduction of blood circulation half-life [20]. Additionally, proteins adsorbed on the surface of nanoceria may also modify their surface chemistry and thus their physicochemical properties, in addition to the hydrodynamic size and the surface charge that determine cellular uptake and subcellular localization [21,22,23].

The goal of this study is the investigation of the possibility of exploiting the advantages of a liposomal drug delivery system in order to increase the therapeutic index of nanoceria. Liposomes are promising vectors that allow for the incorporation of hydrophilic and hydrophobic drugs due to their amphiphilic nature and vescicular structure [24]. Liposomes composed of phospholipids are biocompatible and biodegradable, with a diameter in the range of 50–200 nm, and they generally do not undergo a rapid systemic clearance [24]. Surface modifications by means of polymeric chains as polyethylene glycol (PEG), in fact, provide a stealth layer that reduces their uptake by the immune system, thus prolonging the persistence of the liposomes in the blood circulation [24]. In addition, the use of liposomes can promote a selective delivery of the payload into targeted cells/tissues upon the functionalization of their surface with antibodies, antigens, or small molecules capable of binding specific receptors on the cell membrane [25,26]. Furthermore, such a targeting strategy can be also exploited for the crossing of biological barriers, thus allowing compartments otherwise inaccessible, such as the central nervous system, to be reached [27].

In this work, nanoceria-loaded liposomes were prepared through a reverse-phase evaporation method, extruded with membrane filters, and finally purified with gel permeation chromatography. Obtained hybrid nanovectors were extensively investigated in terms of lipid concentration, size distribution, *Z*-potential, morphology, and loading efficiency. Finally, preliminary in vitro studies have been carried out on normal human dermal fibroblasts to investigate biocompatibility and unaltered antioxidant activity of the entrapped nanoceria.

## 2. Results

### 2.1. Characterization of Liposomes

Empty liposomes and nanoceria-loaded liposomes were prepared by a reverse-phase evaporation method in order to obtain a high loading amount of nanoceria. Size and *Z*-potential were assessed before biological testing, and obtained results are summarized in Table 1. Free nanoceria present an average diameter of 12 ± 1 nm and a *Z*-potential of 32.0 ± 3.8 mV. Empty liposomes and nanoceria-loaded liposomes have a hydrodynamic size of about 165 ± 42 nm and 230 ± 10 nm, respectively. As expected, analyses revealed a negative *Z*-potential of −41.0 ± 11.2 mV for empty liposomes because of the presence of the anionic lipid (1,5-dihexadecyl-*N*-succinyl-l-glutamate, DHSG), while nanoceria-loaded liposomes present a positive surface charge of 30.8 ± 0.4 mV deriving from the adsorption of cerium oxide nanoparticles on the surface of negatively charged liposomes. Values obtained from inductively coupled plasma mass spectroscopy (ICP-MS) revealed 6 mg/mL of complexed nanoceria for a dispersion of 9 mg/mL of nanoceria-loaded liposomes, highlighting a loading efficiency of 12%. The samples were found stable for many weeks since the preparation, resulting into a variation of the hydrodynamic size <15% after one month of storage.

**Figure 1:**
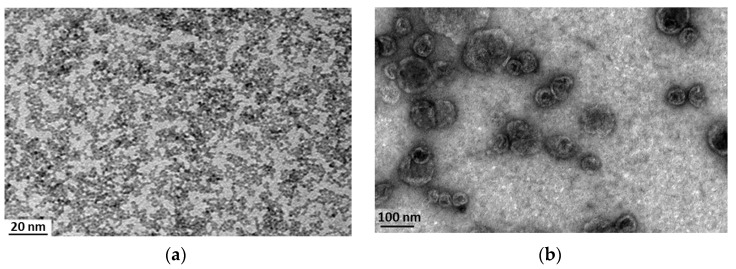
TEM imaging of free nanoceria (**a**) and of nanoceria-loaded liposomes (**b**).

Morphology of the samples was confirmed by transmission electron microscopy (TEM) images. Figure 1a shows the monodispersed cerium oxide nanoparticles used in this work, while Figure 1b highlights the formation of well-defined spherical liposomes, and suggests the presence of cerium oxide nanoparticles complexed to the liposomes, both inside the lipid vesicles and on their surface, as also confirmed by the increment of the *Z*-potential.

The analysis of the total antioxidant capacity (TAC) of nanoceria-loaded liposomes and empty liposomes was assessed and expressed in terms of Trolox equivalents, a water-soluble vitamin E analog that serves as an antioxidant standard [28]. The TAC of 7 µg of nanoceria-loaded liposomes was found significantly higher (6.14 ± 0.34 nmol; * *p* < 0.05) with respect to the negligible effect of the same quantity of empty liposomes (0.09 ± 0.03 nmol), showing strong retained efficiency of the encapsulated nanoceria.

### 2.2. In Vitro Studies

**Figure 2:**
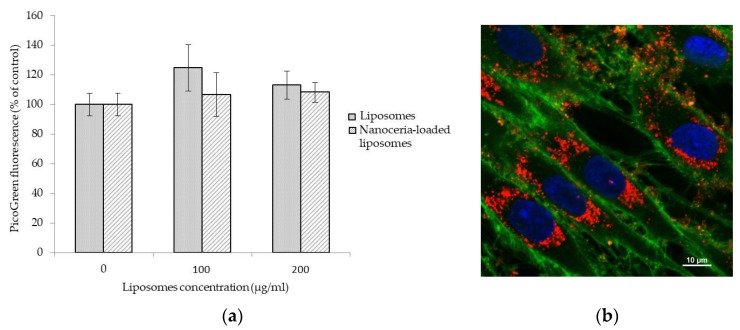
PicoGreen assay on NHDF cells after 48 h of incubation with increasing concentrations of empty liposomes and nanoceria-loaded liposomes (**a**); confocal image showing nanoceria-loaded liposomes (in red) up-taken by NHDF cells after 48 h of incubation; nanoceria-loaded liposomes are stained in red, cell membranes in green, and nuclei in blue (**b**).

Biocompatibility of empty liposomes and of nanoceria-loaded liposomes was investigated on normal human dermal fibroblast (NHDF) cell line by a PicoGreen assay. As shown in Figure 2a, results indicate good cytocompatibility for all the samples up to a concentration of 200 µg/mL and after 48 h of treatment. No significant variation of fluorescence intensity, and therefore of number of cells in the samples, was in fact detected in the different cultures, thus demonstrating no cytotoxic effects of all of the formulations.

Confocal microscopy was instead used to verify the cellular uptake of nanoceria-loaded liposomes, and Figure 2b clearly shows strong internalization by NHDF cells after 48 h of incubation, with a strong accumulation of nanoceria-loaded liposomes (in red) in the perinuclear area of the cells (cell membranes stained in green, nuclei in blue).

**Figure 3:**
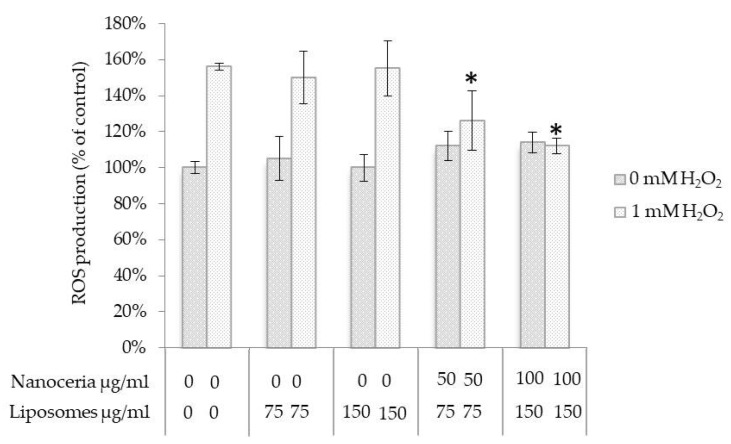
Quantitative evaluation of reactive oxygen species levels in NHDF cells treated with empty liposomes and nanoceria-loaded liposomes, with and without H_2_O_2_ pro-oxidant insult; * *p* < 0.05.

To demonstrate unaltered antioxidant activity of entrapped nanoceria, reactive oxygen species (ROS) formation was assessed. NHDF cells were stimulated with 1 mM H_2_O_2_ for the intracellular generation of ROS, after a pre-incubation with increasing concentrations of nanoceria-loaded liposomes and empty liposomes as a control. Figure 3 shows that the treatment for 45 min with 1 mM H_2_O_2_ induced an increase in the ROS production of about 56% with respect to non-treated cells, which was hindered by the pre-incubation with nanoceria-loaded liposomes in a dose-dependent manner. This demonstrates a maintained antioxidant activity of cerium oxide nanoparticles encapsulated in the liposomes.

## 3. Discussion

The excessive and non-balanced production of free radicals, a process known as oxidative stress, can damage various cell components such as proteins, lipids, and DNA, causing many different diseases [29]. Neurodegenerative disorders, cancer, atherosclerosis, hypertensions, autoimmune diseases, diabetes, and obesity are just some of the pathological conditions associated with oxidative stress [30].

A way to restore the cell redox status could be the use of cerium oxide nanoparticles, the ability of which, as ROS scavengers, has been demonstrated by countless studies in the literature [31,32]. However, though efforts have been made to improve the stability and the biodistribution of nanoceria in vivo through polymeric coatings or surface modifications with ligand moieties, to date only one work reports the use of drug delivery systems as a multi-stage strategy for increasing the therapeutic potential of nanoceria [33]. In this research, the authors obtained cerium oxide nanoparticles encapsulation in poly(lactide-*co*-glycolide) microspheres, without however confirming the ROS scavenging activity of entrapped nanoceria in vitro [33].

As a similar approach, our objective was focused on the investigation of a strategy to load nanoceria into a drug delivery platform, namely, liposomes, that traditionally gained considerable interest in nanomedicine because of many advantageous features. Their high biocompatibility, the possibility of controlling their physicochemical properties, their ability to entrap molecules in both an aqueous core and a lipid bilayer, and, most importantly, the specific targeting to a desired site through an appropriate surface functionalization are only some of the properties that make liposomes an efficient drug carrier [34].

Most of the studies about the encapsulation of small inorganic nanoparticles in liposomes concern quantum dots [35], silica nanoparticles [35], and magnetic nanoparticles [36]. Extensively studied, megnetoliposomes are an interesting example of a multifunctional liposomes/nanoparticles hybrid platform, and they can be exploited as contrast agents in magnetic resonance imaging, for magnetically guided targeting delivery, or for generating heat when exposed to an alternating magnetic field [37].

A study of interaction between liposomes and nanoceria has been approached by Liu et al., who exploited phosphocoline-based liposomes as a model of the cell membrane, but focusing their investigations only on the interaction between nanoceria and biological membranes [38].

In the present work, we demonstrate for the first time that nanoceria can be encapsulated inside liposomes preserving their antioxidant activities. The final lipid composition has been the result of an extensive procedure of optimization, which took into account the stability of the preparation, the encapsulation yield, and the biocompatibility of the nanovectors. Particular attention has also been dedicated to the ease of purification of the final products. The presented formulation was an optimal compromise among all these considered factors.

Thus, nanoceria-loaded liposomes, well-tolerated by fibroblasts, can be extensively internalized by the cells and preserved strong antioxidant activities. The proposed strategy is therefore extremely promising for improving the biodistribution and the in vivo targeting: the encapsulation into liposomes could in fact protect nanoceria by the surface absorption of blood proteins that can affect the interaction with cells, while at the same time protecting the surface chemistry of the nanoparticles and therefore their catalytic activities. Moreover, the PEGylation of the liposome surface and their stability deriving from favorable electrostatic interactions could represent an advantage in overcoming the fast clearance from the blood circulation, by shielding liposomes from immunoglobulins and proteins of the complement system, which act as signals for recognition by the mononuclear phagocytic system. Liposomal lipids could also be used for several strategies of functionalization with targeting moieties, thus avoiding a direct link of ligands on the nanoparticle surface that could hinder their catalytic activities while, at the same time, enabling the targeting and crossing of biological barriers, such as the blood–brain barrier, which represents the major obstacle of nanoceria delivery to the central nervous system. Finally, the capacity of nanocarriers such as liposomes to act as multi-functional vectors for the delivery of multiple cargos offers an interesting stimulus for alternative therapeutic strategies. As an example, co-encapsulation of nanoceria and imaging contrast enhancers such as magnetic nanoparticles could make the liposomes an actual theranostic nanoplatform suitable in all those scenarios where nanoceria can play an important medical action.

## 4. Materials and Methods

### 4.1. Preparation of Lipid Mixture and Liposomes

For the liposome preparation, a lipid anionic mixture of 1,2-dipalmitoyl-sn-glycero-3-phosphocholine (DPPC), cholesterol, 1,5-dihexadecyl-*N*-succinyl-l-glutamate (DHSG), and 1,2-distearoylsn-glycero-3-phosphoethanolamine-*N*-[monomethoxy poly(ethylene glycol) (5000)] (PEG-DSPE) at a molar ratio of 5:5:1:0.03 was used. DPPC and cholesterol were purchased from Nippon Fine Chemicals (Osaka, Japan), PEG-DSPE from NOF Corporation (Tokyo, Japan), and DHSG was in-house synthesized in laboratory [39,40].

A mixture was prepared by dissolving those lipid components (total 100 mg) in *t*-butylalcohol (20 mL); the mixed solution was then freeze-dried overnight, and powder was then used for liposome preparation.

Nanoceria-loaded liposomes were prepared by a reverse-phase evaporation method. More specifically, 10 mg of mixed lipid powder was dissolved in ethanol, and 50 mg of nanoceria was then added (47,232 from Alfa Aesar, Ward Hill, MA, USA), presenting a Ce^3+^/Ce^4+^ ratio of ~1.6, please see characterization reported in Figure S1, Supplementary Materials). After the solvent at 40 °C was removed by rotary evaporation under reduced pressure, the obtained dried lipid-nanoceria mixed film was hydrated and dispersed in 1 mL of water at room temperature for 3 h through stirring.

The liposome dispersion thereafter underwent two extrusion cycles through polycarbonate filters of a 200 nm pore size (Whatman^®^ Nuclepore™ Track-Etched Membranes, Maidstone, UK). In order to separate un-loaded nanoceria from the liposome dispersion, suspension was purified through gel permeation chromatography using a Sephadex G-100 column and deionized water as eluent.

Each fraction containing liposomes was collected and analyzed in terms of size, *Z*-potential, nanoceria content, and lipid concentration. The same procedure was used for empty liposomes preparation, without nanoceria addition. Lipid concentration of the liposomes was calculated from the concentration of cholesterol using a cholesterol assessment kit (Abcam, Cambridge, UK) according to the manufacturer’s instruction.

### 4.2. Liposomes Characterization

Particle size distribution and *Z*-potential of nanoceria, empty liposomes, and nanoceria-loaded liposomes were investigated with a Nano Z-Sizer (Malvern Instrument, Malvern, UK). For both analyses, each measurement was performed three times. Nanoceria content was calculated by means of elemental analysis performed through inductively coupled plasma mass spectroscopy (ICP-MS, Thermo Scientific, Waltham, MA) analysis, and the obtained value was used to determine the encapsulation efficiency through the following equation:

%EE = [(nanoparticles added − free nanoparticles)/nanoparticles added] × 100,(1)

Transmission electron microscopy (TEM) was performed in order to analyze sample morphology. For negative staining, 10 µL of the nanoceria-loaded liposomes dispersion or of free nanoceria solution was placed on carbon-coated 200 mesh copper grids for 5 min and then stained with a 1% uranyl acetate solution. Thereafter, grids were washed three times with water to remove the excess of staining and allowed to air-dry. Transmission electron microscopy of the samples was performed using a JEOL JEM-1011 microscope (JEOL Ltd., Tokyo, Japan) equipped with a tungsten thermionic gun operating at a 100 kV accelerating voltage. TEM images were acquired with a 11 Mp Orius 1000 CCD camera (Gatan, Pleasanton, CA, USA).

The antioxidant efficiency of nanoceria-loaded liposomes was evaluated through a specific assay (the Total Antioxidant Capacity Assay Kit, Sigma, Saint Louise, MO, USA) that allows for the determination of the antioxidant capacity of a substance in terms of Trolox equivalents. Trolox is a water-soluble vitamin E analog [28] and was used as a standard antioxidant in order to obtain a calibration curve following the manufacturer’s instruction. A Cu^2+^ working solution was added to dispersions of empty liposomes, nanoceria-loaded liposomes (in both cases corresponding to an amount of 7 µg of nanovectors), and to all the Trolox standard samples. In the presence of antioxidants, Cu^2+^ ions are converted to Cu^+^ ions, which react with a colorimetric probe giving an absorbance peak at 570 nm. This peak, assessed after 90 min of incubation through a microplate reader (Victor3, PerkinElmer, Waltham, MA, USA), is proportional to the total antioxidant capacity.

### 4.3. Cell Cultures

The normal human dermal fibroblast (NHDF) cell line was purchased from Lonza (Basel, Switzerland) and cultured in Dulbecco’s Modified Eagle’s Medium (DMEM) supplemented with 10% fetal bovine serum (FBS) and 1% penicillin-streptomycin. Cells were grown at 37 °C in an atmosphere containing 5% CO_2_ and passaged by trypsinization with 0.5% trypsin/ethylenediaminetetraacetic (EDTA).

### 4.4. Cytotoxicity of Nanoceria-Loaded Liposomes and Empty Liposomes

The biocompatibility of nanoceria-loaded liposomes and empty liposomes was tested in terms of cell proliferation by the Quant-IT^TM^ PicoGreen^®^ dsDNA assay (Molecular Probes, Eugene, OR, USA), enabling cell number quantification upon evaluation of nucleic acids concentration. Cells were seeded at a density of 6 × 10^3^ cells/cm^2^ in 24-well plates and incubated for 24 h at 37 °C and 5% CO_2_. The medium was then replaced with fresh medium containing empty liposomes or nanoceria-loaded liposomes with different concentrations (0, 100, and 200 µg/mL). After 48 h since the beginning of the treatment, cells were lysed and incubated with the appropriate reagents according the manufacturer’s instructions. Finally, fluorescence was measured in 96-well black plates through a microplate reader (Victor3, PerkinElmer, Waltham, MA; excitation 485 nm, emission 535 nm).

### 4.5. Cellular Uptake Investigation

Internalization of nanoceria-loaded liposomes into NHDF cells was investigated by confocal microscopy. Cells were seeded at a density of 3 × 10^3^ cell/cm^2^ in Ibidi µ-Dishes (35 mm, Ibidi) and after 24 h they were treated with nanoceria-loaded liposomes at a concentration of 100 µg/mL for 48 h. The liposomes were previously stained with a lipophilic red-fluorescent dye (1:200 dilution, Dil, Molecular Probes, Eugene, OR). Finally, cells were rinsed with PBS and treated for 10 min with CellMask, a green-fluorescent dye for labeling cell membranes (1:1000 dilution, Invitrogen, Carlsbad, CA, USA), and with Hoechst 33342 (5 µg/mL, Invitrogen) for nucleus counterstaining. A confocal microscope (C2s, Nikon, Tokyo, Japan) was therefore used for the acquisition of the images.

### 4.6. Intracellular Antioxidant Activity Evaluation

In order to investigate the antioxidant activity of nanoceria-loaded liposomes, 2′,7′-dichlorodihydrofluorescein diacetate (H_2_DCF-DA) Cellular Reactive Oxygen Species Detection Assay Kit (Life Technologies) was used. H_2_DCF-DA is a fluorogenic dye that is able to diffuse through cell membrane and to intracellularly interact with hydroxyl, peroxyl, and other ROS. More specifically, after its cell internalization, H_2_DCF-DA is deacetylated by cellular esterases to a non-fluorescent compound, which is later oxidized by ROS into 2′,7′-dichlorofluorescein (DCF), a fluorescent compound that thus becomes a direct indicator of the intracellular ROS levels. NHDF cells were seeded in a 24-well plate at a density of 6 × 10^3^ cell/cm^2^, incubated for 24 h, and then treated for 48 h with dispersions of empty liposomes or nanoceria-loaded liposomes at different concentrations (0, 75, and 150 µg/mL), respectively containing 0, 50, and 100 µg/mL of nanoceria. In order to induce intracellular ROS accumulation, cells were treated with 1 mM H_2_O_2_ for 45 min at 37 °C in a complete medium, and finally incubated with 100 μM H_2_DCF-DA in a serum-free medium at 37 °C in the dark for 30 min. After cell lysis through three freezing/thawing cycles, fluorescence was quantified with a microplate reader (Victor3, Perkin Elmer, Waltham, MA; excitation 485 nm, emission 535 nm).

### 4.7. Statistical Analyses

Data were analyzed with one-way ANOVA followed by Bonferroni’s post hoc test or two-tailed unpaired *t*-test through KaleidaGraph (Synergy Software). In all experiments, performed in triplicate, data with a *p-*value < 0.05 were considered statistically significant.

## 5. Conclusions

Liposomes were demonstrated to be an excellent platform for nanoceria encapsulation without altering antioxidant properties of the nanoparticles. Nanoceria-loaded liposomes showed good biocompatibility, a regular spherical shape, nanometric size, and preserved antioxidant activity in vitro. Further investigations will be needed in order to modify the liposome surface with appropriate ligands for a targeted delivery. The encapsulation of nanoceria into a liposomal drug delivery system could improve the pharmacokinetic profile of nanoceria in vivo and represents a stimulus for new strategies of nanotechnology-based therapies.

## Figures and Tables

**Table 1:** Characterization of nanoceria, liposomes, and nanoceria-loaded liposomes dispersions.

Sample	Size (nm)	Polydispersity Index	*Z*-Potential (mV)
Nanoceria	12 ± 1	0.156 ± 0.010	32.0 ± 3.8
Empty liposomes	165 ± 42	0.147 ± 0.006	−41.0 ± 11.2
Nanoceria-loaded liposomes	230 ± 10	0.263 ± 0.023	30.8 ± 0.4

## Supplementary Materials

The following are available online at http://www.mdpi.com/2079-4991/7/9/276/s1, Figure S1: XPS analysis of free nanoceria.
