# Supplementary material for: Preparation, Characterization, and Preliminary In Vitro Testing of Nanoceria-Loaded Liposomes
Source: Nanomaterials (Basel). 2017 Sep 16;7(9):276. doi: 10.3390/nano7090276 (PMC5618387; doi:10.3390/nano7090276)
Supplement: Supplementary file 1 [file nanomaterials-07-00276-s001.docx]

Preparation, characterization, and preliminary *in vitro* testing of nanoceria-loaded liposomes

Agostina Grillone*, Tianshu Li, Matteo Battaglini, Alice Scarpellini, Mirko Prato, Shinji Takeoka, and Gianni Ciofani*

**Supplementary materials**

Materials and Methods

X-ray photoelectron spectroscopy (XPS) analysis has been carried out to assess Ce^3+^ and Ce^4+^ content. Samples have been prepared by pressing a few milligrams of finely ground CeO_2_ nanoparticles powder onto a high purity In pellet (Sigma Aldrich). Measurements have been carried out using a Kratos Axis Ultra^DLD^ spectrometer (Kratos Analytical Ltd., UK) using a monochromatic Al Kα source (hν = 1486.6 eV) operated at 20 mA and 15 kV. The analyses have been carried out on 300 × 700 μm area. Wide scans have been collected at pass-energy of 160 eV and energy step of 1 eV, while high-resolution spectra have been collected at pass-energy of 10 eV and energy step of 0.1 eV. The Kratos charge neutralizer system has been used for all the measurements. Spectra have been analyzed with CasaXPS software (Casa Software, Ltd., version 2.3.17).

Results

Figure S1 shows the results of the XPS data collected on the energy region typical for Ce 3d peaks after background subtraction, together with the outcome of the fitting procedure. The best fit has been obtained with five sets of spin-orbit split doublets, three of which representative of Ce(IV) (green profiles in the figure) and two of Ce(III) (blue profiles) oxides, as described in reference [1]. The splitting between the two components of each doublet has been set to 18.6 eV, as reported in reference [2] while the intensity ratio between the two components is set to 3:2, due to spin-orbit coupling.


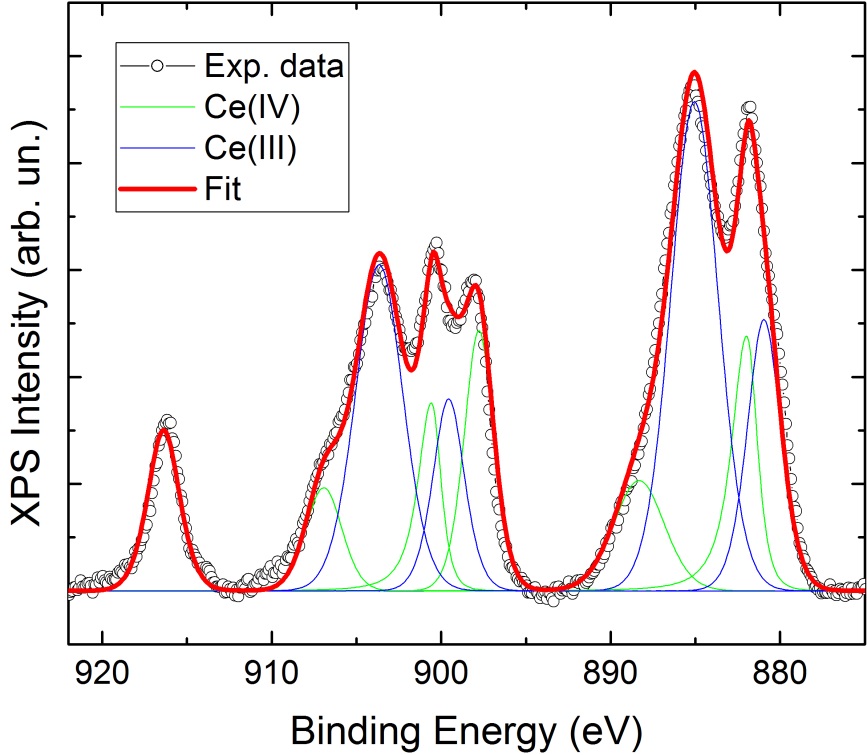


Figure S1. XPS analysis of free nanoceria.

The percent concentration of Ce^3+^ (Ce^4+^) ions in the sample has been calculated from the ratio of the sum of the integrated areas of the XPS 3d peaks related to Ce^3+^ (Ce^4+^) to the total integral area for the whole Ce 3d region. The acquired XPS spectrum is consistent with a Ce^3+^ percent concentration of (61±2) % (Ce^4+^ percent concentration of (39±2) %), corresponding to a Ce^3+^/Ce^4+^ ratio of ~1.6.

Supplementary References

1. Zhou, Y.; Perket, J.M.; Zhou, J. Growth of Pt Nanoparticles on Reducible CeO_2_(111) Thin Films: Effect of Nanostructures and Redox Properties of Ceria. *J. Phys. Chem. C* **2010**, *114*, 11853–11860, doi**:**10.1021/jp1007279.

2. Beche, E.; Charvin, P.; Perarnau, D.; Abanades, S., Flamant, G. Ce 3d XPS investigation of cerium oxides and mixed cerium oxide (Ce_x_Ti_y_O_z_). *Surf. Interface Anal.* **2008**, *40*, 264–267, doi:10.1002/sia.2686.
